# Navigating urban ecology: Conceptual and methodological insights

**DOI:** 10.1002/ecy.70402

**Published:** 2026-06-05

**Authors:** Ian MacGregor‐Fors, Christine C. Rega‐Brodsky, D. Johan Kotze, J. Scott MacIvor, Amy K. Hahs, Marco Moretti, Nicholas S. G. Williams, Christopher A. Lepczyk, Kirsten Jung, Madhusudan Katti, Sonja Knapp, Frank A. La Sorte, Max R. Piana, Caragh G. Threlfall, Paige Warren, Charles H. Nilon, Myla F. J. Aronson

**Affiliations:** ^1^ Department of Environmental Sciences, Faculty of Biological and Environmental Sciences University of Helsinki Helsinki Finland; ^2^ School of Natural Resources University of Missouri Columbia Missouri USA; ^3^ Department of Biological Sciences University of Toronto Scarborough Toronto Ontario Canada; ^4^ School of Agriculture, Food and Ecosystem Sciences The University of Melbourne Richmond Victoria Australia; ^5^ Swiss Federal Research Institute WSL Biodiversity and Conservation Biology Birmensdorf Switzerland; ^6^ College of Forestry, Wildlife and Environment Auburn University Auburn Alabama USA; ^7^ Evolutionary Ecology and Conservation Genomics Ulm University Ulm Germany; ^8^ Department of Integrative Humanities and Social Sciences North Carolina State University Raleigh North Carolina USA; ^9^ Department of Community Ecology UFZ—Helmholtz‐Centre for Environmental Research Halle (Saale) Germany; ^10^ German Centre for Integrative Biodiversity Research (iDiv) Halle‐Jena‐Leipzig Leipzig Germany; ^11^ Department of Ecology and Evolutionary Biology Yale University New Haven Connecticut USA; ^12^ Center for Biodiversity and Global Change Yale University New Haven Connecticut USA; ^13^ Harvard Graduate School of Design Harvard University Cambridge Massachusetts USA; ^14^ School of Natural Sciences Macquarie University Macquarie Park New South Wales Australia; ^15^ Department of Environmental Conservation University of Massachusetts Amherst Massachusetts USA; ^16^ Department of Ecology, Evolution and Natural Resources Rutgers, The State University of New Jersey New Brunswick New Jersey USA

**Keywords:** interdisciplinarity, research methodology, social‐ecological systems, urban biodiversity, urban ecology, urbanization

## Abstract

Since its consolidation as a sub‐discipline of ecology in the late 1990s, urban ecology has grown substantially, enhancing our understanding of how urbanization shapes biodiversity and how biodiversity adapts to urbanization. Inherently interdisciplinary in its scope and application, urban ecology is rooted in multiple theoretical and methodological foundations, potentially making the field overwhelming for newcomers as the key literature is scattered across multiple disciplines. Drawing on the collective expertise of editors and reviewers, we attempt to address common pitfalls noted in studies of biodiversity within urban ecosystems and suggest action items for both the new and experienced urban ecologist. Here, we present key concepts for framing hypothesis‐based urban biodiversity research questions, conceptualizing urban settings, accounting for spatial and temporal scales, selecting study systems and meaningful explanatory variables for research questions, and recognizing the social‐ecological nature of urban systems. We offer insights and examples, emphasizing the importance of rigorous urban ecological research without imposing constraints on innovation.

## INTRODUCTION

Urban ecology rose to prominence in the late 1990s, but the discipline is rooted in research dating back to the mid‐19th century (Goode et al., 2020; Kowarik, 2020; Nilon, 2023; Sukopp, 2008). Since its inception, ecologists have adapted existing knowledge and practices to establish the theoretical and methodological foundations of urban ecology (Forman, 2016; Niemelä, 1999). These theories and methods have evolved and been refined as urban ecology has matured into an interdisciplinary field of research (Flaminio et al., 2023; Forman, 2016; Pickett & Cadenasso, 2017). This evolution has led to a surge of studies that enhance our understanding of how urbanization and its related social‐historical factors affect biodiversity, as well as how biodiversity responds to these changes (Aronson et al., 2014; Johnson & Munshi‐South, 2017; McDonnell & Hahs, 2015; Nilon, 2023; Rega‐Brodsky et al., 2022).

Urban ecology encompasses four key paradigms that are all currently relevant (Byrne, 2022; Pickett et al., 2016, 2022). Ecology *in* the city examines ecological patterns and processes in often applied studies within urban areas (e.g., Slabbekoorn & Peet, 2003). Ecology *of* the city frames entire cities as integrated social‐ecological systems (e.g., Hope et al., 2003). Ecology *for* the city focuses on integrating ecological knowledge to improve urban design and development (e.g., Krause et al., 2023), while ecology *with* the city emphasizes co‐production with communities to address social justice and livability (see Knapp et al., 2021 for examples). Although we present the paradigms in the chronological sequence they were proposed, the paradigms represent complementary and mutually reinforcing perspectives that can be combined depending on research goals and context. *In*/*of* studies continue to provide foundational insights, while *for*/*with* approaches highlight opportunities for intervention and participatory engagement.

Thus, the study of urban ecosystems, encompassing these paradigms and intersecting multiple disciplines, has drawn a diverse range of researchers, including ecologists previously focused on other ecosystems. This convergence of basic and applied sciences and scientists underscores the necessity of incorporating all actors across spatiotemporal scales (McDonnell & MacGregor‐Fors, 2016; Pickett et al., 2016). However, the complexity of urban systems presents challenges for engaging in meaningful research (Pickett et al., 2020), which can lead to studies with theoretical, methodological, and interpretative shortcomings. To contribute to overcoming these limitations and to improve comparability and interpretation across urban ecology studies, we have identified common challenges, with a special focus on biodiversity research, and provide insights and examples for both newcomers and experienced researchers. We do not seek to criticize, prescribe, or limit innovation; rather, we aim to offer guidance based on our experience. While not all studies will be able to implement every recommendation, applying them progressively, whenever possible, could help build a more cohesive understanding of urban ecology and strengthen research across regions.

## CRAFTING IMPACTFUL HYPOTHESIS‐BASED RESEARCH QUESTIONS INCORPORATING THE COMPLEXITY OF URBAN SYSTEMS

Articulating clear, hypothesis‐based research questions is essential across all fields. Urban ecology is no exception, as it requires well‐defined frameworks to unravel the complex interactions between social, historical, and ecological factors. Unique phenomena—such as the urban heat island effect, where urban areas experience elevated temperatures compared to non‐urban surroundings, especially at night due to reduced cooling, while daytime differences remain smaller because of similar solar irradiation—highlight the need to rethink, and even reframe, some ecological principles to urban contexts (Forman, 2016; Pickett & Cadenasso, 2017). This foundational step requires researchers to address the distinct ecological dynamics of urban systems. Equally critical is a comprehensive familiarity with both the classic and contemporary urban ecology literature, including seminal books and reviews (e.g., Berkowitz et al., 2003; Forman, 2014; Nilon & Aronson, 2023), which together provide the conceptual and empirical foundations upon which new studies are built. Despite advancements, many studies still do not fully engage with existing research or address critical knowledge gaps, as they have traditionally focused on describing patterns rather than underlying processes (Rega‐Brodsky et al., 2022). While replicating research and using standardized methodologies enhances our understanding of patterns in understudied regions, researchers should also aim to advance the discipline through mechanistic, experimental, and theoretical approaches (Grilo et al., 2022; Shochat et al., 2006).

When framing research questions, hypotheses, and predictions, researchers could benefit by drawing upon the main urban ecology paradigms mentioned above, while also testing their assumptions in new ways, such as through comparative studies, experimental interventions, novel metrics, or participatory approaches that evaluate their applicability across different urban contexts. Furthermore, additional frameworks have been proposed that focus on particular aspects of *in*, *of*, *for*, and *with* paradigms (Byrne, 2022; Childers et al., 2015; Pickett et al., 2016), offering opportunities to design hypothesis‐based empirical studies that further our understanding of the mechanisms driving urban ecological systems. Studies grounded in the ecology *in* and *of* the city paradigms provide foundational observational insights that inform, motivate, and contextualize more mechanistic, experimental, or participatory approaches. At the same time, studies and action based on the ecology *for* and *with* the city expand the discipline by integrating broader social‐ecological dimensions and by testing how ecological mechanisms operate across urban contexts. By linking more mechanistic frameworks to the ecology *in* and *of* the city paradigms, researchers can design studies that not only test specific ecological mechanisms but also address broader conceptual questions about how urban systems function and respond to interventions. For example, researchers can explore how environmental, ecological, socio‐economic, and cultural filters shape species occurrence and abundance using urban community assembly frameworks (Aronson et al., 2016). While much of the current evidence is correlational, these patterns allow researchers to formulate mechanistic hypotheses that can be tested in future empirical or experimental studies (McDonnell & Hahs, 2013; Shochat et al., 2006).

Urban ecology also presents opportunities to test general ecological theories, such as community assembly and biodiversity–ecosystem function relationships. Over the past 10–15 years, process‐oriented research, mostly focused on the mechanisms underlying ecological patterns at the individual, population, and species levels, including behavioral, physiological, adaptive, and evolutionary processes, has expanded significantly. This growth has advanced our understanding of urban ecological dynamics within the broader field of ecology (Knapp et al., 2021).

## CONCEPTUALIZING AND CHARACTERIZING THE URBAN SETTING

To ensure ecological studies are reproducible and comparable, researchers must clearly define the characteristics of their study systems (MacGregor‐Fors, 2011; McDonnell & Hahs, 2013). Urban ecosystems, in particular, are characterized by small‐scale spatial land‐use heterogeneity (Forman, 2016), high prevalence of non‐native species, and a large proportion of private land (Loram et al., 2007; Richardson et al., 2025). While these features require careful consideration to accurately characterize urban environments and enable meaningful comparisons, it is important to acknowledge that some essential variables (e.g., management, nutrient inputs, human use of greenspaces) may be difficult to quantify in practice, in certain contexts. For instance, management intensity may vary among private landowners and is rarely systematically documented; nutrient inputs can be diffuse, episodic, or derived from multiple sources (e.g., fertilizers, pet waste, atmospheric deposition), complicating direct measurement.

### Global, regional, and local characteristics of urban study areas

Describing the study region, from landscape context to specific sampling sites, is crucial for understanding the research context and ensuring reproducibility and comparability across studies (e.g., Hahs, 2023). While differing urban landscapes may share common features, cities exhibit significant variability due to biogeographic, historical, and cultural differences (du Toit et al., 2021), and conditions within them can change over time and space through both anthropogenic (e.g., urban construction, reorganization) and non‐anthropogenic factors (e.g., climatic, hydrological, ecological processes). Compounding this complexity, terms used in the literature to describe urban classifications (e.g., “suburban,” “rural”) may have different meanings globally. For instance, the term “suburban” may indicate wealthy areas in some regions (e.g., USA, Western Europe), but poverty in others (e.g., Latin America; Rega‐Brodsky et al., 2026), and the meaning of “rural” greatly varies across regions (MacGregor‐Fors & Vázquez, 2020). These differences can lead to unexpected findings, such as contrasting biodiversity trends with increasing urbanization (e.g., Rega‐Brodsky & MacGregor‐Fors, 2023) or conflicting patterns driven by the use of categorical descriptions of urban intensity rather than quantitative continuous measures of the level of urbanization (Caryl et al., 2014).

Understanding the spatial distribution of a city's greenspace network and its historical legacy is crucial for further understanding of cities, as they evolve over time with inherent heterogeneity (Forman, 2016). Two sites with similar developed, built‐up land cover (referred to as built cover hereafter) may differ ecologically based on time since development, often influencing biodiversity (Munyenyembe et al., 1989). Historical processes may shape greenspace patch size, as well as their connectivity and land use (Helm et al., 2006; Johnson et al., 2018). These legacies, shaped by urban growth rates, can influence present‐day human demographic and cultural patterns, which in turn directly affect habitat structure, species distribution, and human–nature interactions (du Toit et al., 2021). Robust urban biodiversity assessments may thus benefit from incorporating these factors to both contextualize and shape research questions, hypotheses, and variable selection, even if they are not the primary focus of the study (Turrini & Knop, 2015).

To effectively describe urban study regions and sampling sites, we suggest including the following features: the climatic or bioregional zone and surrounding ecosystems, as well as basic information about the urban settlement (e.g., size, spatial structure, dominant urban land‐use types, characteristic patterns of human activity directly linked to the built environment). When reporting the size of the settlement, we recommend avoiding government boundaries and focusing instead on spatially aggregated urban land uses (Lemoine‐Rodríguez et al., 2019; MacGregor‐Fors, 2011). For multicity approaches, a comparative table with consistent and reliable data sources is highly recommended (e.g., Falfán et al., 2018).

### Framework and scale for accurately describing urban locations

Urban classifications and intensities vary within and between cities, requiring consistent definitions for accurate analysis and interpretation (MacGregor‐Fors, 2011; Moll et al., 2019). The classic concentric zone theory (Park & Burgess, 1925) has often been used, where urban intensity is inferred by distance from the city core (e.g., Dallimer et al., 2012; Schoeman, 2015). However, this model does not reflect how most cities grow. A more effective approach quantifies the proportion of built cover within a relevant distance from a survey location, tailored to the ecology and behavior of the studied taxon (McDonnell & Hahs, 2013).

Several studies illustrate how the spatial scale and historical context of urban locations influence ecological interpretations. For example, research focusing on bird–habitat associations has examined landscape characteristics at different extents (e.g., from 1 km^2^ areas to smaller radii of 25–100 m), demonstrating that ecological patterns may shift depending on the spatial frame of reference (Escobar‐Ibáñez et al., 2020; Marzluff et al., 2001). It is also important to note that a substantial proportion of greenspaces within cities have been designed, created, or managed during the urbanization process. Although these areas are technically part of the urban fabric, under certain conditions they can function as valuable habitats (Fairbairn et al., 2024). Therefore, understanding the origin, legacy, and composition of urban greenspaces (e.g., primary vegetation remnants, agricultural plots, horticultural gardens, urban‐industrial vegetation; Kowarik, 1992) is crucial for accurately situating ecological observations within an appropriate spatial and historical framework.

### Reporting urbanization levels accurately and reproducibly

Urban intensity is typically measured by the proportion or percentage of built cover in two dimensions, which includes the sum of constructed surfaces such as roads, parking lots, and buildings. A value near 100% indicates full urbanization, while 0% reflect non‐built areas, often covered by vegetation or other non‐artificial surfaces (Rega‐Brodsky et al., 2022). Studies often reveal a strong negative relationship between built cover and biodiversity, with most indicating a decline in species richness as urban intensity increases (e.g., bird communities; Rega‐Brodsky & MacGregor‐Fors, 2023). However, some studies show a hump‐shaped or even a positive relationship, depending on the scale and sampling design (Concepción et al., 2016; Escobar‐Ibáñez et al., 2020).

While these traditional two‐dimensional measures have been useful, they overlook the vertical complexity of cities, which can strongly influence ecological patterns. Although some studies have considered building height in urban survey sites (e.g., Melo et al., 2022), recent advancements allow researchers to incorporate three‐dimensional information, including the use of light detection and ranging (LiDAR) to quantify the structural arrangement of buildings and vegetation (Lepczyk et al., 2021). This added dimensionality can improve our understanding of wildlife responses and adaptations to urban life, as many species interact with the urban matrix in inherently vertical ways. For example, aerial insectivore birds and bats often depend on open flight corridors between buildings, while canopy‐foraging birds respond to the vertical layering of vegetation (Stewart & Oke, 2012). By moving beyond traditional 2D metrics, LiDAR‐based approaches reveal the structural heterogeneity of urban systems, where boundaries between urban and non‐urban areas can be functionally ambiguous (MacGregor‐Fors, 2010; United Nations, 2005).

Several variables have been used as proxies of urbanization levels across landscapes. Common examples include human population density and environmental pollution, such as artificial light at night (La Sorte et al., 2022). Other studies have focused on anthropogenic noise or vehicle traffic as proxies, particularly when examining the effects of urban noise on animal behavior (Carral‐Murriera et al., 2020; Parris et al., 2009; Warren et al., 2006). In addition to selecting appropriate proxies, some researchers apply statistical methods to summarize multiple variables into a single urbanization metric. For instance, principal components analysis (PCA) can be used to derive an urbanization axis that integrates several indicators (Casanelles‐Abella et al., 2024). This approach can be complex to interpret because it combines multiple variables, which may obscure the influence of individual factors and make results context‐dependent. Simpler univariate proxies such as population density or artificial light at night are easier to interpret but capture only a single dimension of urbanization. Each approach thus involves trade‐offs between interpretability, accuracy, and applicability for management or planning.

Quantifying urbanization levels using continuous variables is generally more suitable for precise analyses than relying on discrete categories such as “urban,” “suburban,” or “rural.” However, when categorical classifications are used, providing clear and quantifiable definitions for terms with inherently variable interpretations (e.g., rural, suburban; MacGregor‐Fors & Vázquez, 2020; Rega‐Brodsky et al., 2026) is essential to ensure consistency across studies and to facilitate future synthesis and meta‐analyses. While categorical approaches may be appropriate in certain theoretical or methodological contexts, they have limitations (García‐Arroyo, Gómez‐Martínez, et al., 2025). First, categorizing urbanization levels—such as labeling areas where the quantification of built cover is 70% as “high” in different cities—may lead to misrepresentations due to varying urban characteristics like land cover and population density. Second, categorical variables may obscure the relationship between urbanization and ecological outcomes, as continuous variables allow for a more accurate assessment of these dynamics (Caryl et al., 2014). Categorical classifications, particularly when they are overly broad, can obscure meaningful variation in urban characteristics and ecological responses. Using coarser categories to describe urbanization intensity, for instance, may mask subtle changes in species responses or habitat use, while finer categories can reveal more nuanced relationships. This highlights that the choice of category resolution can influence how urban effects are interpreted (García‐Arroyo, Gómez‐Martínez, et al., 2025).

To improve comparability and interpretation, we strongly advise that researchers use continuous quantitative measures of urbanization level whenever possible. Alternatively, internationally standardized classification frameworks and procedures, such as those proposed by Lososová et al. (2011) for comparing biodiversity across European cities, could offer a more unified approach. These frameworks standardize the way urban habitats or land‐use types are categorized, the resolution of spatial units, and the criteria for measuring biodiversity, facilitating comparisons across different cities and studies while reducing inconsistencies caused by locally specific classifications. However, no universal classification scheme currently exists to address the full range of urban contexts worldwide. Notably, while standardized approaches can help structure available data, it is important to recognize that cities differ in their history, morphology, and social‐ecological dynamics, and thus comparisons across urban contexts should be interpreted with caution.

As mentioned earlier, it is crucial not only to measure urbanization levels accurately but also to report the methods used for quantification to ensure reproducibility in urban ecology studies, avoiding vague “rural‐to‐urban” gradient definitions (Hahs, 2023; MacGregor‐Fors & Vázquez, 2020). McDonnell and Hahs (2008) found that only five out of 300 reviewed studies quantified urbanization level, a trend that persists today (Rega‐Brodsky et al., 2022), making synthesis and generalization difficult. For instance, Williams et al. (2015) had to rely on a vote‐counting approach, tallying whether studies report “positive,” “negative,” or “neutral” effects of urbanization on plant traits or urban flora composition, due to methodological variability in urban ecology studies, whereas Vaz et al. (2023) had to categorize data from diverse urbanization intensity gradients into broad categories (i.e., “light,” “suburban,” and “urban”; without providing explicit definitions, at least not evidently in the manuscript) to try to harmonize studies on insect diversity, while also grouping a wide range of what they termed “informal greenspaces” (e.g., gardens, city parks, small urban parks). Similarly, Batáry et al. (2018) standardized inconsistent land‐use classifications into “natural,” “rural,” “suburban,” and “urban” categories based on study descriptions, and in cases where only qualitative descriptions were given, they accepted or reclassified categories as needed, but emphasized that in some cases, it remained difficult to clearly separate urban and suburban classes.

Adding to these challenges, the spatial heterogeneity and temporal changes in cities deserve careful consideration (Ossola et al., 2021; Weng, 2007), as sites that superficially appear similar may not always be replicated in terms of environmental and social‐ecological conditions. For instance, in a study examining migratory bird habitat use along a forest fragment to residential land use transect, birds exhibited distinct habitat preferences across the interior and edges of different land uses (Buron et al., 2022). This highlights the importance of accounting for land‐use and land‐cover heterogeneity, as edge effects and the proximity of adjacent land uses can strongly influence biodiversity. Furthermore, human activity, including fluctuations in traffic levels and differences between weekdays and weekends, can influence data and should be considered (Filazzola et al., 2022). To support reproducibility and comparability across studies, providing coordinates where permissible can be helpful, but this alone does not capture the full variability of urban sites. Therefore, it is important to complement georeferencing with detailed descriptions of site characteristics and local conditions.

### Selecting explanatory variables beyond urbanization level

When studying biodiversity patterns in urban systems, researchers often go beyond urbanization levels to consider factors such as built structure characteristics, vegetation traits, human activities, economy, demographics, pollution, and urban hazards (Moll et al., 2019). Effectively capturing these variables would enable a detailed understanding of the complexity of these social‐ecological systems and shape study outcomes. While no universal approach exists, certain key considerations can help ensure research efforts yield meaningful insights. One important consideration is using ecologically meaningful scales when selecting independent variables, as biodiversity metrics can vary with spatial grain and extent (Haase et al., 2012). Additionally, because ecological patterns often reflect taxon‐specific habitat availability at spatial levels (Beninde et al., 2016), incorporating site‐scale variables that capture conditions most relevant to the focal organisms is essential. The relative importance of these factors can vary considerably across taxonomic groups, and therefore considering all variables potentially relevant to their focal taxa is recommended, including, for instance, vegetation (e.g., vegetation cover, structure, and composition), human activity levels (e.g., pedestrians, passing vehicles), potential hazards (e.g., predators, light and noise pollution), or socioeconomic and historical land‐use factors (Hope et al., 2003; MacGregor‐Fors & Schondube, 2011). Doing so not only strengthens the ecological accuracy of urban studies but also enhances their practical value, informing habitat management and urban planning efforts (Garden et al., 2006).

Additionally, reviewing existing literature, particularly reviews and meta‐analyses, can help researchers identify standardized methodologies and avoid limitations associated with inconsistent approaches, while still allowing room for incorporating new variables measured in novel ways. However, the lack of comparability and transparency in past studies has often hindered the ability to conduct robust meta‐analyses.

### Recognizing variety in urban greenspace type, structure, and function

When studying urban greenspaces, it is essential to provide a detailed description of their biological structure and composition (Aronson et al., 2017). These spaces harbor distinct biotas influenced by factors such as regional species pools, landscape and environmental characteristics, management practices, and cultural functions like recreation (La Sorte et al., 2023; Threlfall et al., 2017). Greenspaces classified under the same name (e.g., gardens, cemeteries) can exhibit significant variation across cultures, countries, and biomes, with differences in structure. For example, the structures of cemeteries in Italy and German‐speaking Switzerland tend to differ, with varied densities in Italy and more park‐like structures in Switzerland (Moretti, pers. comm.). Even within a single category, conditions may vary, impacting biodiversity. For example, urban forests may contain different forest types, species, age structures, and conditions, influencing the types of habitats and biota present (Piana et al., 2021). Additionally, it is important to consider ownership and governance type. Individual, privately owned greenspaces often receive narrower management strategies compared to public ones, which are more frequently managed to accommodate a broader range of recreational activities and amenities (Dyson et al., 2019; Loram et al., 2008; Thompson et al., 2003). These factors underscore the need for comprehensive descriptions to understand biodiversity and inform management and planning efforts.

## MATCHING TAXONOMIC GROUPS AND ORGANIZATIONAL LEVELS WITH HYPOTHESES

Matching taxonomic groups with the hypotheses to be tested is essential for understanding how and why biodiversity declines, persists, or thrives in urban environments (Fischer et al., 2015). Urban areas filter the regional biota pool, which means certain taxonomic groups may be less informative as study systems, or even absent across urban environments (Aronson et al., 2016; Fournier et al., 2020; MacGregor‐Fors, García‐Arroyo, & Quesada, 2022). Thus, “indicator species” that are representative of environmental quality features (Morelli et al., 2021) or “umbrella species” that indicate high taxonomic and functional diversity (Sattler et al., 2014) may be the best matches to certain hypotheses.

The chosen taxon and the level of biological organization (e.g., genes, individual organisms, populations, communities) should not only align with the research hypotheses but also reflect the specific biota of the urban setting in question. Research questions within the realm of adaptation and evolution may center on genetic to population levels (Diamond & Martin, 2021; Johnson & Munshi‐South, 2017), while questions exploring behavioral plasticity may focus on individual to population levels (Sol et al., 2013). Studies examining human impacts on biodiversity in cities often span multiple levels, from genes and phylogeny (Miles et al., 2019) to communities (Rega‐Brodsky et al., 2022) with a particular focus on species assemblages.

When selecting taxonomic groups and organizational levels based on the scope of a hypothesis, it is important to consider the origin of species or taxa. For example, in urban studies, plants can be assessed according to whether they are intentionally planted or spontaneous, which may influence the observed ecological patterns and responses (Figueroa et al., 2018; Nagase et al., 2019). Similarly, non‐native and invader species are often analyzed separately from native or non‐invader species, following biological invasion science practices (García‐Arroyo, Rega‐Brodsky, & MacGregor‐Fors, 2025; Van Kleunen et al., 2010). However, studies should include all species—native, non‐native, invader, spontaneous, or cultivated—unless there is a specific justification for excluding them, as urban landscapes often feature novel species assemblages with potentially important ecological roles (Francis & Chadwick, 2015). Moreover, urban environments tend to favor species that exhibit high adaptability or plasticity to anthropogenic conditions, which can influence biodiversity patterns (Fischer et al., 2015; MacGregor‐Fors et al., 2010; McDonnell & Hahs, 2015). Thus, considering all species allows for a comprehensive understanding of urban biodiversity.

## ADDRESSING CHALLENGES IN FIELD DATA COLLECTION, DESIGN, AND SAMPLING APPROACHES

An essential part of conducting some urban ecology research projects is the collection of field data, which presents unique challenges in urban environments. Access to study sites may be limited by private land, gated communities, or industrial areas, and researchers often face transportation limitations, lack of roads, or security risks in areas with known criminal activity (Ramírez‐Restrepo et al., 2015). Careful attention must be paid to the logistics of fieldwork, particularly when using expensive or specialized equipment, which is at higher risk of theft or tampering in urban settings (López‐Flores et al., 2009). Additionally, personal safety risks can be amplified for individuals with minority identities (Demery & Pipkin, 2021). To mitigate these challenges, researchers are encouraged to proactively familiarize themselves with study sites and engage with local communities and authorities. Securing permission, conducting site visits, informing the community, wearing visible safety gear, and carrying institutional identification are essential to ensure safe and effective fieldwork (Dyson et al., 2019). These precautions require careful planning and preparation and should be explicitly acknowledged to avoid biases in data collection.

Building on these considerations, frameworks and survey schemes have been used to test urban ecology hypotheses across different scales, from local to citywide and multicity perspectives. Local studies have been critical in shaping our understanding of urban ecology, especially regarding urban environmental impacts, such as fragmentation, isolation, and pollution (Rega‐Brodsky et al., 2022). Notably, their findings can carry meaningful weight for local policy and management. For example, a study on wild bee diversity in Toronto informed the development of the Pollinator Protection Strategy, which included community grants, changes in park management, and planting practices. This initiative, led by local decision makers and city authorities, shows how ecological research can guide practical urban conservation measures and serve as a model for other cities (City of Toronto, 2019).

As highlighted above, urban systems can be singular due to interacting factors that shape their spatial configurations and habitat suitability for species, which together create highly heterogeneous and novel environmental conditions rarely found in non‐urban areas (Graham et al., 2017; Plummer et al., 2020). This uniqueness is reflected in the wide variation in social, ecological, and built characteristics within cities, which influences species composition and individual species (Alberti & Wang, 2022). Urban biodiversity is often concentrated within the city's greenspace network, which includes remnants of natural and historical human‐made habitats as well as carefully designed and managed gardens and urban‐industrial ecosystems (Aronson et al., 2017; Kowarik, 1992; Trigos‐Peral et al., 2020). These areas differ in connectivity, management, and exposure to urban pressures, emphasizing the need to account for this heterogeneity when testing ecological hypotheses or controlling for confounding factors in field studies.

When establishing survey sites within urban settings or within their regions, an additional aspect to consider is their spatial location. Regardless of the approach used to characterize urbanization, ecological isolation driven by the urban matrix remains an important factor. The proximity of study sites to the city's geographic center or its boundary, for example, can impact biodiversity responses in terms of ecological isolation, especially in large cities (MacGregor‐Fors & Ortega‐Álvarez, 2011). Studies indicate that local heterogeneity becomes crucial at small scales, whereas at larger scales, the location within cities, such as peri‐urban areas, can override other well‐known drivers of urban biodiversity (Evans et al., 2009; MacGregor‐Fors, 2010; Puga‐Caballero et al., 2014).

Scaling up from local studies, more research is being conducted at citywide or multicity levels (Schmit et al., 2025), sometimes with a global perspective (Hahs et al., 2023; La Sorte et al., 2018; Rega‐Brodsky et al., 2022). Citywide schemes require a representative sample of urban conditions, taking into account the uneven distribution of land‐use types across cities (Falfán et al., 2018; MacGregor‐Fors et al., 2012; Turner, 2003). Stratified random sampling based on land use, urban intensity, and environmental conditions can help capture this diversity. These approaches improve our understanding of urbanization levels and allow spatially explicit comparisons with other urban elements (MacGregor‐Fors, Falfán, et al., 2022; Schell et al., 2020). For multicity analyses, it is advisable to account for differences among cities, including their inherent characteristics, as well as potential biases, such as observer and geographic biases, to ensure accurate comparisons (MacGregor‐Fors et al., 2020; Rega‐Brodsky et al., 2022).

Opportunistic experiments are common in cities due to urban growth, policy complexities (e.g., water management), or site selection based on development over time (Katti et al., 2017). However, manipulative study designs are also necessary to better understand the drivers of urban biodiversity, beyond correlates (McDonnell & Hahs, 2013; Shochat et al., 2006). Such experiments are rare in urban ecology due to challenges in city environments, with exceptions like studies on urban residents’ exposure to nature, which are mostly observational (>80%, Marvier et al., 2023; Shanahan et al., 2015). While observational studies are valuable, they may lead to misleading conclusions. For example, a meta‐analysis of 109 studies showed that positive trends in pollinator and plant species richness across land‐use types could not be confirmed in manipulation studies (Kral‐O'Brien et al., 2021).

## INCORPORATING COLLABORATIVE STRATEGIES FOR BUILDING ETHICAL RESEARCH ACROSS URBAN ECOSYSTEMS

Cities are hubs where scientists, planners, citizens, and other actors converge and collaborate (Byrne, 2022). As spaces where both basic and applied sciences intersect, they highlight the importance of incorporating the perspectives and values of all relevant groups across spatiotemporal scales (Pickett et al., 2016). Pickett et al. (2022) emphasized this convergence through their concept of the co‐production of place (within the ecology *with* the city paradigm), which underscores that urban areas are shaped by ongoing planned and unplanned development, influencing the biological, physical, and social dimensions of the environment.

Collaborating with local scholars and community members, particularly Indigenous groups when present, is essential for initiating urban ecology projects. These groups bring valuable place‐based knowledge, historical insights, and a deeper understanding of that city's history (Trisos et al., 2021). Such partnerships not only enrich research outcomes but also foster mutual learning and enhance understanding of urban biodiversity and its ecological and cultural value (Knapp et al., 2021; Tanner et al., 2014; Trisos et al., 2021). However, maintaining strong, long‐term collaborations with place‐based partners often requires being mindful of how much we ask of them. Not every research question needs direct community involvement; certain topics can be explored from a disciplinary perspective alone and still provide insights that are valuable both locally and across broader urban ecology contexts. Undoubtedly, involving local communities fosters respect and trust, crucial for managing urban ecosystems effectively and creating inclusive partnerships that enhance the impact of urban ecology research (Childers et al., 2015).

Urban ecology studies often extend beyond biodiversity to include site‐related variables, which require the collaboration of multidisciplinary teams with regional or global representation (Knapp et al., 2021). Increasing recognition of the importance of social science partnerships has highlighted the need to integrate human‐centered variables—both perspectives and values—into urban ecological research (McPhearson et al., 2016). Collaborative efforts are also evident in participatory science (citizen science) initiatives, where large‐scale “big data” is collected, benefiting both researchers and practitioners (e.g., La Sorte et al., 2026).

Global and regional research networks are invaluable for addressing large‐scale research questions that are difficult to test in isolation. This is particularly relevant as we work to understand the ecology *in*, *of*, *for*, and *with* cities (Byrne, 2022; Pickett et al., 2016). Multicity collaborations are especially beneficial for comparative research, data sharing, and fostering interdisciplinary engagement (McPhearson et al., 2016). Several established urban ecological networks offer opportunities for participation and growth, such as the Urban Biodiversity Research Coordination Network‐UrBioNet (Aronson et al., 2016; https://sites.rutgers.edu/urbionet), CitiesWithNature (https://citieswithnature.org), Urban Biodiversity Hub (https://ubhuborg.wixsite.com/aboutus), the Urban Wildlife Information Network (Magle et al., 2019; https://www.urbanwildlifeinfo.org), or the recently created Urban Bird Consortium‐URBICON.

## NAVIGATING THE PATH AHEAD

Here, we explore key conceptual and methodological aspects for the future of urban ecological research, with a particular focus on guiding those new to the field, including early‐career researchers and ecologists transitioning from other systems, while also offering a useful point of reflection for more experienced urban ecologists. This paper aims to assist this audience in navigating the rapidly evolving field of urban ecology, while also advocating for greater standardization of methods and data reporting. By doing so, it not only enhances our understanding of urban environments, where the majority of the global population resides, but also facilitates future comparative studies and meta‐analyses by providing high‐quality, comparable data. In Box 1, we present a checklist to assist in planning future urban ecology studies. It is important to note that this checklist is a guide, not a strict reference, and some items may not apply to every project. As urban ecological research advances, new conceptual challenges will emerge alongside rapidly evolving methodologies. By acknowledging these challenges and offering some guidance on how to navigate them, we aim to contribute to the continued development of urban ecology as a vital field for understanding and managing the complexities of urban environments.

BOX 1 **Checklist of questions to address prior to conducting a study to avoid common pitfalls in urban biodiversity research. Some only apply to specific research questions and approaches.**

**Crafting impactful hypothesis‐based research questions incorporating the complexity of urban systems**

1. Have the hypotheses been placed within an urban ecology framework?
2. Have ecological principles or paradigms been considered within the context of urban ecology?

**Conceptualizing and characterizing the urban setting**

1. Has well‐defined terminology been applied to describe the study sites?
2. Has the historical legacy influencing urbanization at the sites been considered?
3. Have the spatial extent, geographic location, population size, physical extent, and primary land uses of the urban setting been documented?
4. Have the major ecosystems adjacent to the focal cities or towns been described?
5. Has the climatic or bioregional zone of the urban area under study been acknowledged?
6. Has a quantitative/continuous or qualitative/categorical/discrete measure been used to describe urbanization levels?
7. Is the description of how urbanization levels were determined sufficiently detailed to ensure reproducibility?
8. Was the urbanization level measured within the timeframe of the study?
9. Have relevant site‐scale variables (e.g., vegetation traits, human activity levels, socioeconomic and historical land‐use factors, pollution, potential hazards) been incorporated beyond urbanization levels in the study design?
10. Have urban greenspaces been characterized in sufficient detail, including their vegetation structure, composition, management, and governance?
11. Have general urban classifications (e.g., suburban, rural, exurban, peri‐urban) been described in detail?

**Matching taxonomic groups and organizational levels with hypotheses**

1. Have the taxonomic groups and organizational levels been appropriately matched to the hypotheses under investigation?
2. Has the origin (native or non‐native) of the study taxon and any interacting organisms been identified?

**Addressing challenges in field data collection, design, and sampling approaches**

1. Has the spatial scale been appropriately selected to best address the research question (e.g., local, citywide, multicity, regional)?
2. Have the geolocations of study plots/sites been reported whenever possible?
3. Should a manipulative experimental design be implemented to answer the focal research question?
4. Have ecologically meaningful scales been considered to best address the research question (e.g., local, citywide, multicity, regional), accounting for the location of study sites within the urban setting and potential biases such as observer and geographic biases?
5. Are all field sites accessible, or is permission required for access?
6. Have personal safety considerations in the field been thoroughly evaluated?

**Incorporating collaborative strategies for building ethical research across urban ecosystems**

1. Have local leaders, scholars, and Indigenous groups, when present, been considered and included in the project?
2. Have the potential impacts of the study, including sampling and other research activities, on the local community been considered?
3. Can local or regional communities contribute to and/or benefit from the project?
4. Are there collaborative networks addressing similar questions or providing data that could enhance the research?

## CONFLICT OF INTEREST STATEMENT

The authors declare no conflicts of interest.
